# Biooxidation of a Pyrite-Arsenopyrite Concentrate Under Stressful Conditions

**DOI:** 10.3390/microorganisms12122463

**Published:** 2024-11-29

**Authors:** Aleksandr Bulaev, Alena Artykova, Anna Diubar, Aleksandr Kolosoff, Vitaliy Melamud, Tatiana Kolganova, Alexey Beletsky, Andrey Mardanov

**Affiliations:** Research Center of Biotechnology, Russian Academy of Sciences, 119071 Moscow, Russia; alena.artykov@gmail.com (A.A.); annadbr1@yandex.ru (A.D.); alexander_thechemist_kolosoff@mail.ru (A.K.); vmelamud.inmi@yandex.ru (V.M.); moldiag@biengi.ac.ru (T.K.); mortu@yandex.ru (A.B.);

**Keywords:** biohydrometallurgy, acidophilic microorganisms, pyrite, arsenopyrite, sulfide concentrates, stressful conditions, carbon dioxide

## Abstract

Gold recovery from refractory pyrite-arsenopyrite concentrates using stirred tank reactor biooxidation is widely applied worldwide. Therefore, studies to address the characteristic problem of this technology are urgent. The goal of the present work was to research the possibility of counteracting the negative effects of unfavorable conditions (increasing pulp density and temperature) on the biooxidation of pyrite-arsenopyrite concentrate in laboratory-scale stirred tank reactors using additional carbon supply in the form of CO_2_. A refractory concentrate containing pyrite (48%) and arsenopyrite (13%) was used in biooxidation experiments. In the control experiment, biooxidation was performed under “normal conditions”: temperature 40 °C, pulp density (solid to liquid ratio, S:L) 1:10, residence time 5 days. It was shown that under “normal conditions”, additional carbon dioxide supply insignificantly affected the biooxidation rate and composition of the microbial population of biooxidation reactors. In addition, the effect of “stressful conditions” was studied. In this case, either temperature or pulp density were increased (up to 50 °C and S:L 1:5, respectively), which provided unfavorable conditions for biooxidation and led to the decrease in biooxidation rate. Under “stressful conditions”, additional carbon dioxide supply affected biooxidation to a greater extent and made it possible to increase both pyrite and arsenopyrite biooxidation rates. The analysis of microbial populations showed that additional carbon dioxide supply also changed their composition.

## 1. Introduction

Gold recovery from a refractory pyrite-arsenopyrite concentrate can be increased using stirred tank reactor biooxidation (STRB), which is widely applied worldwide [1,2,3]. STRB has some advantages over other technologies (roasting, pressure oxidation); it does not require high energy consumption and complex equipment, and allows one to avoid toxic gas emissions when arsenic-containing concentrates are processed [1,2,3,4,5].

The base of the biohydrometallurgical processing of a refractory concentrate is sulfide mineral biooxidation by acidophilic iron- and sulfur-oxidizing microorganisms. The simplified overall reactions may describe the biooxidation of sulfide minerals, components of gold-bearing concentrates (arsenopyrite, pyrite) [6]:

2FeAsS + 7O_2_ +H_2_SO_4_ + 2H_2_O→2H_3_AsO_4_ + Fe_2_(SO_4_)_3_ (1)

4FeS_2_ + 15O_2_ + 2H_2_O→2Fe_2_(SO_4_)_3_ + 2H_2_SO_4_ (2)

According to Equations (1) and (2), sulfide mineral biooxidation leads to the dissolution of iron ions and arsenic, as well as pH reductions due to sulfuric acid formation.

The temperatures of industrial bioleaching processes are usually in the range of 40–45 °C due to heat production during mineral biooxidation and certain difficulties of performing biooxidation processes using microorganisms with a higher temperature optimum. Thus, the prevailing groups in microbial populations of biooxidation reactors are thermotolerant and moderately thermophilic acidophilic microorganisms. According to numerous studies, STRB is always performed by mixed populations of different acidophilic microorganisms including several species of iron- and sulfur-oxidizing microorganisms [7,8,9,10,11,12,13,14,15,16,17,18]. The composition of the oxidized concentrate, temperature, pH, oxygen, and carbon availability affect the composition of the microbial consortia [3].

A predominance of different thermotolerant and moderately thermophilic acidophilic microorganisms was shown by the analysis of microbial populations formed under both the industrial-scale and the laboratory-scale bioleach reactor modeling conditions applied in industrial processes, which may be explained by the elevated temperature maintenance. It was found that bacteria of the genera *Leptospirillum* and *Sulfobacillus*, moderately thermophilic bacteria of the genus *Acidithiobacillus* (*A. caldus*), as well as archaea of the family *Ferroplasmaceae* (genera *Acidiplasma* and *Ferroplasma*) were predominant microorganisms in these microbial populations [7,8,9,10,11,12,13,14,15,16,17].

Thus, microorganisms of various taxonomic groups with different properties ordinarily compose the microbial populations of the reactors. These populations include the following:

- Microorganisms capable of oxidizing either ferrous iron (Fe^2+^) or reduced inorganic sulfur compounds (RISC), as well as ones oxidizing both Fe^2+^ and RISC;
- Microorganisms with different types of carbon nutrition including autotrophs, which fix dissolved carbon dioxide using the energy obtained by the ferrous iron and RISC oxidation, and mixo- and heterotrophs, which require organic carbon sources for stable growth, besides the fact that they also gain energy by the oxidation of inorganic compounds [1,2,18].

As was mentioned above, the temperature of the pulp of biooxidation reactors increases due to exothermic chemical reactions. Over-heating may inhibit microbial activity; therefore, the temperature of industrial-scale reactor pulp is maintained using a cooling system [3,5,6].

The carbon supply of acidophilic microorganisms is one of the key factors that may control the activity of sulfide mineral biooxidation in both the reactors and natural ecosystems, and is partially determined by microbial interactions in the populations [3,5,6,19,20].

The results of some previous research show that the addition of extra carbon sources (organic or inorganic) facilitates the intensification of the biooxidation process of sulfide concentrates, impacts the composition of microbial consortiums, and allows one to decrease the inhibitory effects of negative factors, for example, increasing pulp temperature [20,21,22].

Both organic and inorganic carbon sources were shown to enhance the biooxidation rate and affect the microbial population, causing an increase in the total number of microorganisms and changes in the composition of the populations [20,21,22]. At the same time, the biooxidation activity depends on the use of additional carbon sources to a greater extent at higher temperatures. For example, experiments on the biooxidation of pyrite-arsenopyrite concentrate at 40 and 50 °C in batch mode using CO_2_ have demonstrated that at both temperatures, the additional carbon dioxide supply affected the biooxidation rate. However, at 50 °C, this effect was more significant than at 40 °C [21]. Thus, additional carbon supply may also be considered as a method to decrease the negative effects of bioleached reactor pulp over-heating.

In the work of Bulaev et al. [20], the biooxidation of a pyrite-arsenopyrite concentrate was performed in continuous mode in the temperature range from 40 to 50 °C using carbon dioxide and molasses as carbon sources. Temperature increase was performed stepwise (40–45–50 °C), and for biooxidation in continuous mode at each temperature, the microbial population was adapted to biooxidation in a batch mode. A higher temperature led to a lower rate of biooxidation, but the application of additional carbon dioxide as a carbon source prevented the inhibition of the process.

Thus, the results of our previous works demonstrate that additional carbon supply (for example, in the form of carbon dioxide) may help avert the effects of inhibitory factors affecting the biooxidation of sulfide concentrates, namely, increasing temperature.

The main goal of the present work was to study the effect of carbon dioxide supply on the biooxidation of pyrite-arsenopyrite concentrate under unfavorable conditions (temperature and pulp density increase). In contrast to previous studies, in the present study we performed an increase in the studied parameter values without stepwise adaptation of the population to the increase. This increase allowed us to study the effect of the stress caused by the shift of temperature and pulp density both on the biooxidation activity and the composition of microbial population. In the present work, we used a consortium of acidophilic microorganisms to perform biooxidation tests. It is known that industrial biooxidation processes are performed by microbial consortia [7,8,9,10,11,12,13,14,15,16,17,18,19,20,21,22]. This is due to several factors, primarily the fact that industrial processes in the mining industry cannot be performed in such a way as to provide the stable functioning of pure culture. Also, microbial consortia of several species may facilitate the robustness of biooxidation process during changes of the conditions (temperature, concentrate composition, and other parameters), as these consortia include several species with different optimum growth conditions. Moreover, interspecies interactions in these consortia are of high interest. Therefore, the present article, as well as several of our previous works [20,21,22], studied microbial consortia, as well as their transformations caused by changes in biooxidation conditions. Thus, in the present work, we have analyzed the transformation of the acidophilic microbial consortium caused by different stress factors (temperature and pulp density increase) in order to understand possible adaptive shifts in its composition.

## 2. Materials and Methods

### 2.1. Concentrate

In the experiments, we used a refractory concentrate containing pyrite (48%), arsenopyrite (13%), quartz, muscovite, sulfide iron (Fe_s_)—26.9%, sulfide arsenic (As_s_)—6.0%, and sulfide sulfur (S_s_)—25.1% (Tables S1 and S2 and Figure S1). 

### 2.2. Experimental Setup and Biooxidaton

In the research, we used technological parameters similar to those used in industrial-scale reactors (the temperature, pulp density and residence time), i.e., “normal conditions”, and “stressful conditions”, which included increased temperature and pulp density, which in turn had negative effects on bio-oxidation.

**Figure 1:**
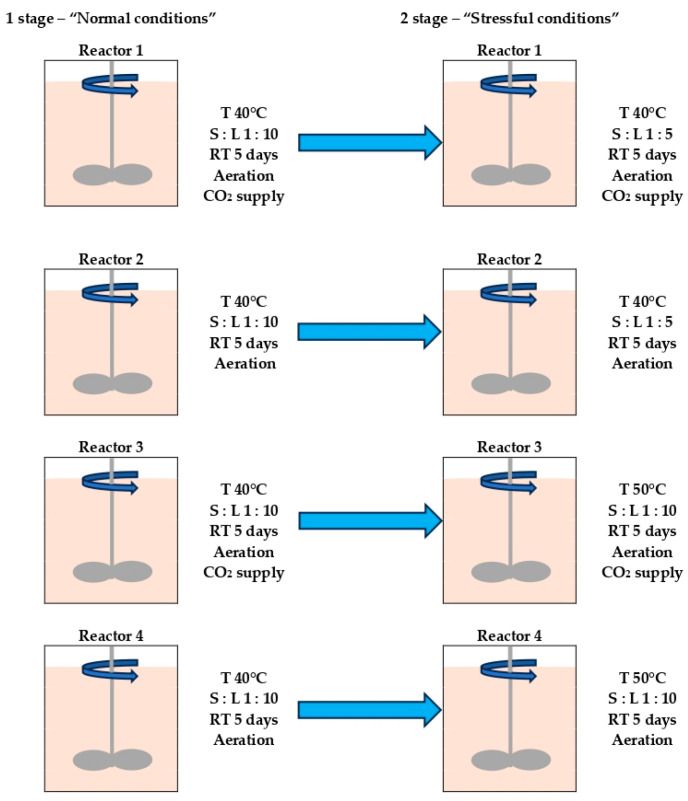
Scheme of the experiment.

The process of concentrate biooxidation was conducted in continuous mode in 4 laboratory-scale stirred tank bioreactors with working volumes of 1.0 L in parallel. A scheme of the experiment is shown in Figure 1. In the first stage, all parameters of the experiment were the same (“normal conditions”). In the second stage (“stressful conditions”), after conducting experiments under “normal conditions”, pulp density was increased in reactors 1 and 2, while temperature was increased in reactors 3 and 4.

In all variants of the experiment, retention time (RT) was 5 days, the speed of turbine mixer rotation was 500 rpm, and aeration rate was 5 L/min. Pulp temperature was maintained using ELMI TW-2.03 circulating water baths (Elmi, Riga, Latvia) and U-shaped titanium heat exchangers. RW-20 digital overhead stirrers (IKA, Staufen, Germany) were used for stirring.

Pulp density (solid to liquid ratio, S:L) was 1:10 (100 g of concentrate per 1000 mL of liquid phase) under “normal conditions”. Under “stressful conditions”, it was increased to S:L 1:5 (200 g of concentrate per 1000 mL of liquid phase) in the reactors 1 and 2, while in the reactors 3 and 4, it was 1:10. Under “normal conditions”, the temperature in all reactors was 40 °C, while under “stressful conditions”, the temperature was increased up to 50 °C in the reactors 3 and 4, while in the reactors 1 and 2, it was 40 °C (Figure 1).

The biooxidation of sulfide minerals of the concentrate (Equations (1) and (2)) is an exothermic process. Heat generation during sulfide biooxidation has been considered in detail in [5]. At the same time, a real increase in temperature takes place in industrial-scale reactors, which have working volumes of several tens to thousands of cubic meters. Due to heat release, the temperature in industrial reactors should be maintained using cooling systems to avoid harmful temperature increases. In laboratory-scale reactors, despite heat release during sulfide mineral oxidation, temperature is not really increased due to the small volumes (up to several liters). Therefore, in this case, temperature in laboratory-scale reactors is maintained at temperatures close to those used in industrial practice (40–45 °C) [5] using various thermostats. Therefore, in our tests, we performed biooxidation at 40 °C (“normal conditions”) and 50 °C (“stressful conditions”) maintaining these temperatures using circulating water baths with heat exchangers.

Pulp temperature was maintained using ELMI TW-2.03 circulating water baths (Elmi, Riga, Latvia) and U-shaped titanium heat exchangers. RW-20 digital overhead stirrers (IKA, Staufen, Germany) were used for stirring.

Reactors 1 and 3 were supplemented with additional CO_2_ (approximately 0.01 L/min) using U-30P gas pressure reducers (Brima, Suzhou, China) to evaluate the effects of additional CO_2_ on biooxidation both under “normal” and “stressful” conditions. Reactors 2 and 4 were not supplemented with CO_2_ (control reactors), and carbon dioxide supplied with aeration was the lone carbon source in this case.

A liquid nutrient medium used for experiments contained the following (g/L of distilled water): (NH_4_)_2_SO_4_—0.75, KCl—0.05, MgSO_4_ × 7H_2_O—0.125, and K_2_HPO_4_—0.125 [20]. The initial pH (1.4) was adjusted by adding 3 mL/L of concentrated sulfuric acid to the medium. During biooxidation, the pH was adjusted by adding either 98% concentrated sulfuric acid or CaCO_3_ to the medium. Sulfuric acid or CaCO_3_ may be used to decrease or increase pH value, respectively.

A microbial population formed during the continuous biooxidation of a similar sulfide concentrate at 40–50 °C, which was used as inoculum [20]. For this purpose, a mixed pulp sample from the reactors operating at different temperatures (40, 45, 50 °C) was used. Prior to the experiment, this sample was incubated in the reactor at 40 °C, S:L 1:10, and an initial pH value 1.5, with the same concentrate and liquid medium as those used in the present study, for 20 days (other parameters were not controlled during the cultivation). After the cultivation, the following parameters were reached: pH = 1.25, Eh = 838 mV, Fe^3+^ concentration 27.3 g/L, Fe^2+^ concentration 0.07 g/L, total cell number 4.8 × 10^7^ cell/mL. Prior to the biooxidation test performed in the present work, the composition of the inoculum was determined using the high-throughput sequencing of 16S gene V3-V4 variable fragments of the 16S rRNA gene (See Section 2.4), and the predominance of the bacteria *Acidithiobacillus* and *Sulfobacillus*, as well as archaea of the genus *Ferroplasma*, were shown (Table S3).

Prior to biooxidation in continuous mode under “normal conditions”, biooxidation was performed in a batch mode to provide adaptation of the inoculum and microbial growth. For this purpose, microbial biomass was inoculated in the reactors containing 1 L of the nutrient medium with pH of 1.4 and 100 g of the concentrate (initial cell number was ~1.2 × 10^7^ cell/mL). Then, the microbial population was grown at 40 °C with aeration and CO_2_ supply as described above. The microbial growth and biooxidation of a pyrite-arsenopyrite concentrate in batch mode was evaluated using the liquid phase parameter (See Section 2.3) and performed until the parameters of the liquid phase stopped changing. Then, the biooxidation was switched to continuous mode under “normal conditions”. After performing biooxidation under “normal conditions”, the conditions were switched to “stressful” ones without an additional adaptation period. In total, biooxidation was performed for 22 days in batch mode, while under “normal conditions” in continuous mode, biooxidation was performed for 25 days, and under “stressful conditions” in continuous mode, biooxidation was performed for 14 days (Figure S2).

### 2.3. Sampling and Analysis

To analyze the activity of the bioleaching, samples of the liquid phase were collected. In all samples, pH and redox potential (Eh) were determined using a pH-150MI pH meter (Izmeritelnaya tekhnika, Moscow, Russia), and ferrous and ferric iron concentrations were measured by the titration with Trilon B (EDTA 2 Na) [23]. Arsenic concentration was determined using a Kvant-2mt atomic absorption spectrometer (Cortec, Moscow, Russia). The quantitative assessment of microorganisms was carried out by direct counts using an Amplival (Carl Zeiss, Jena, Germany) microscope equipped with a phase-contrast device. 

After the biooxidation, the solid residues were separated from the liquid phase of the pulp, dried, and analyzed to determine the oxidation state of sulfide minerals. The chemical composition of solid samples including Fe, As, and S content, was determined using an ARL Perform’x Sequential X-ray fluorescence spectrometer (Thermo Fisher Scientific, Waltham, MA, USA). The preparation of the samples to determine sulfide iron (Fe_s_), arsenic (As_s_), and sulfur (S_s_) was performed according to the phase analysis method (Table S2) [24]. The mineral compositions of the concentrate and biooxidation residues were determined by use of a Rotaflex D/MAX-RC diffractometer (Rigaku, Tokyo, Japan).

### 2.4. Microbial Population Analysis

The composition of microbial populations that formed during the experiments under different conditions was assessed by the metabarcoding of V3-V4 variable fragments 16S rRNA gene fragment using the MiSeq system (Illumina, San Diego, CA, USA). The procedures of biomass sampling, DNA extraction, amplification, sequencing, and bioinformatic analysis were described in detail in our previous work [20]. In short, 16S rRNA gene fragments reads of all samples were clustered together into OTUs (operational taxonomic unit) at 97% identity, and the relative abundance of each OTU at each sample was calculated by mapping reads to the OTUs. Low-quality reads, singleton OTUs and chimeric sequences were removed during the process.

To evaluate the effects of biooxidation conditions on the microbial population, inoculum was sampled before the experiment, and biomass samples were also collected in the end of batch mode, as well as in the continuous mode (under both “normal” and “stressful” conditions). In continuous modes, samples of biomass were collected twice in each variant of the experiment.

The 16S rRNA gene V3-V4 fragment sequences were deposited in the NCBI Sequence Read Archive and are available via the BioProject accession number PRJNA1166071.

## 3. Results

### 3.1. Liquid Phase Analysis

Table 1 shows average liquid phase parameters under “normal conditions”. The presented data demonstrate that in this case, parameters differed insignificantly between reactors with and without carbon dioxide addition, which suggests that carbon dioxide supply had an insignificant effect on biooxidation.

Table 2 shows average liquid phase parameters under “stressful conditions”. In contrast to “normal conditions”, the presented data demonstrate that in this case, parameters differed significantly between reactors with and without carbon dioxide addition. At the same time, a shift of the parameters resulted in the inhibition of biooxidation.

In the pair showing normal/increased pulp density, pH increased, Eh decreased, Fe^3+^ and Fe_tot_ dropped and Fe^2+^ increased in the reactor without carbon dioxide addition, with increasing pulp density. Arsenic concentration increased by 1.5 times when increasing pulp density by 2-fold. In comparison, in the reactor with carbon dioxide supply, the pH and Eh values, as well as ferric iron concentration, differed insignificantly under “normal/stressful conditions”. Ferrous iron concentration increased by 10 times, but it was 4 times lower than that in the control reactor without carbon dioxide supply. Arsenic concentration was 1.8 higher than under “normal conditions”. It should be noted that in the control reactor, ferric iron concentration increased by 10 g/L (1.4 times) in the first 5 days after a shift of pulp density, but then it steadily decreased to 11 g/L (Figure S2). In contrast, in the reactor with CO_2_ supply, ferric iron concentration decreased in the first days after pulp density shift, but then it steadily increased (Figure S2). This suggests the selection of a microbial population after the pulp density increased when using carbon dioxide supply.

In the pair with normal/increased temperature, temperature shift and carbon dioxide supply also affected liquid phase parameters. The pH value increased in both reactors, while in the reactor with carbon dioxide supply, the pH increase was less significant. The Eh value and ferric iron content decreased in both reactors. At the same time, the ferric iron concentration in the reactor with dioxide supply decreased by about 2.3 times, while in the reactor without carbon dioxide supply, the ferric iron content diminished by 5-fold. The ferrous ion concentration in the pulp of the reactor with dioxide supply was 1.5 g/L (6.9 times lower than the concentration of ferric iron), while in the control reactor, the ferric/ferrous ion ratio was 1.85. In contrast to the experiment with an increase in pulp density, in the case of a temperature increase, the ferric iron concentration steadily decreased in both reactors. At the same time, the rate of ferric iron concentration decrease was lower when using additional carbon dioxide.

Thus, in both experiment, CO_2_ supply affected liquid phase parameters under “stressful conditions”. 

### 3.2. Solid Residue Analysis

The comparison of solid residue confirmed the trend revealed by liquid phase analysis (Table 3). Table 3 shows that under “normal conditions”, arsenopyrite oxidation was close to 97% in all bioreactors, with or without carbon dioxide addition. At the same time, pyrite oxidation differed between bioreactors with and without carbon dioxide addition by about 6%.

Under “stressful conditions”, arsenopyrite oxidation was the highest in the experiment with a pulp density increase and with carbon dioxide supply (89.8%, i.e., 7.6% lower than under “normal conditions”). In this case, pyrite oxidation was also the highest for biooxidation under “stressful conditions” but significantly lower than that under “normal conditions” (45.7%, i.e., ~1.8 times lower than under “normal conditions”). In the control reactor, when increasing pulp density, the level of mineral oxidation was lower than those in the reactor with CO_2_ supply (by 1.3 and 1.9 times for arsenopyrite and pyrite, respectively, i.e., 1.4 and 3.2 times lower than under “normal conditions”). 

In the experiment with increasing temperature, the arsenopyrite oxidation was lower than that in the experiment with a pulp density increase, and 1.5 and 1.7 times lower than that under “normal conditions” in the reactor with and without CO_2_ supply, respectively. Pyrite oxidation was slightly higher than in the control reactor with increased pulp density, but the level was 2.6–3.1 times lower than that under “normal conditions” and 1.6–1.7 times lower than in the reactor with CO_2_ supply at increased pulp density.

Thus, the analysis of solid biooxidation residues revealed that CO_2_ supply affected the biooxidation of pyrite under “normal conditions”, while arsenopyrite oxidation reached ~97% regardless CO_2_ supply. At the same time, at increased pulp density, the oxidation of both arsenopyrite and pyrite depended on CO_2_ supply. Increasing the temperature inhibited both pyrite and arsenopyrite biooxidation. At the same time, at 50 °C, the effect of CO_2_ supply on mineral biooxidation was insignificant.

It should be noted that the pH values in all continuous experiments were low (Table 1 and Table 2) and CaCO_3_ was used to regulate the pH, while sulfuric addition was not required. The CaCO_3_ consumption shown in Table 3 corresponds to sulfide mineral oxidation, as it is used for the neutralization of the sulfuric acid formed during biooxidation (Equation (2)).

### 3.3. Microbial Population Analysis

**Figure 2:**
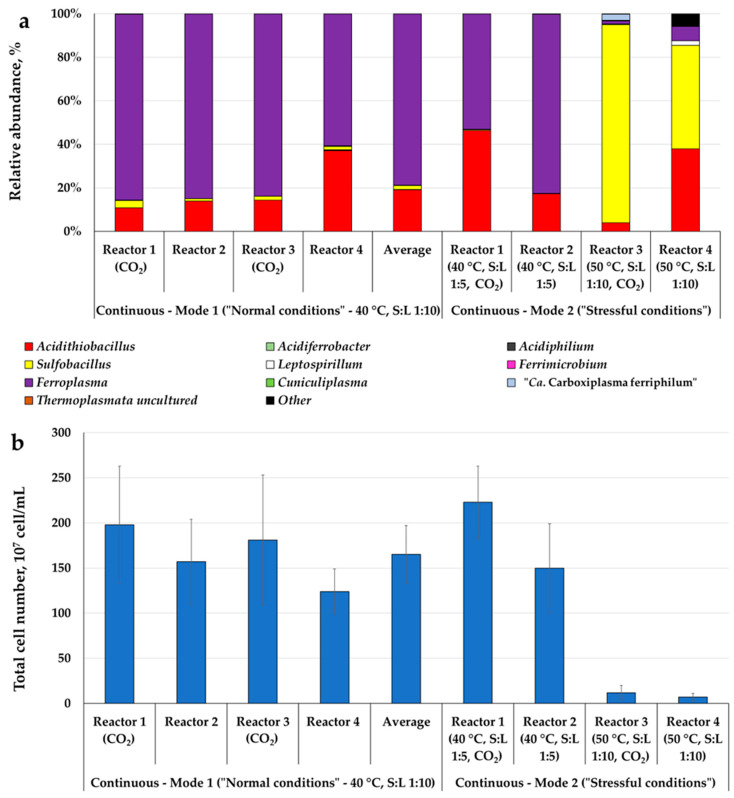
An analysis of the microbial populations of bioleach reactors formed under different conditions: (**a**) a proportion of the 16S rRNA gene fragment, %; (**b**) the total cell number in the liquid phase of the pulp, 10^7^ cell/mL (average cell number ± SD, *n* = 5).

A shift in biooxidation conditions led to a change in the composition of the microbial population (Figure 2 and Table S3).

Under “normal conditions”, bacteria of the genus *Acidithiobacillus* and archaea of the genus *Ferroplasma* were predominant, while bacteria of the genus *Sulfobacillus* were detected in all reactors, but the fraction did not exceed 3.4%. The fraction of other microbial groups was less than 1%. CO_2_ supply had no significant effect on the population composition.

The same groups were predominant when increasing pulp density, while the fraction of the genus *Sulfobacillus* decreased. The total cell number also did not change significantly after the pulp density increase.

Increasing temperature significantly changed the composition of the microbial population. The total cell number decreased by 15–20 times. Bacteria of the genus *Sulfobacillus* were predominant in both reactors with and without CO_2_ supply. The fraction of the genus *Acidithiobacillus* decreased but was significant. Bacteria of the genus *Leptospirillum*, as well as archaea of the genus *Ferroplasma*, *Cuniculiplasma*, and uncultivated archaea “*Ca.* Carboxiplasma ferriphilum” [20], were detected.

## 4. Discussion

A comparison of the results obtained with those published in previous papers on biooxidation pyrite-arsenopyrite concentrate allows us to increase our understanding of the patterns of the biooxidation of gold-bearing sulfide concentrates. Moreover, novel results on the effect of the carbon source on biooxidation were obtained.

As carbon nutrition is one of the key factors affecting the activity of microorganisms applied in biohydrometallurgy, this aspect has been actively explored [18]. It is known that populations of acidophilic microorganisms oxidizing sulfide ores and concentrates include autotrophs (which fix dissolved carbon dioxide), heterotrophs (which require organic carbon sources for stable growth), and mixotrophs (which both fix dissolved carbon dioxide and use organic carbon sources) [18]. The carbon assimilation pathway in acidophilic microorganisms oxidizing sulfide minerals have been identified [18,25]. Microorganisms detected in microbial populations in the present work (Figure 2, Table S3) have different types of carbon nutrition and different carbon dioxide fixation pathways. For example, bacteria of the genera *Acidithiobacillus* and *Sulfobacillus* fix carbon dioxide via the Calvin Cycle, while bacteria of the genus *Leptospirillum* use reversed tricarboxylic acid cycle for autotrophic fixation (despite the genomes of some representatives of the genus *Leptospirillum* including genes encoding RubisCO subunit) [18,25,26]. At the same time, known representatives of *Acidithiobacillus* and *Leptospirillum* are autotrophs, while *Sulfobacillus* are mixotrophs [18,24]. Closely related iron-oxidizing archaea of the genera *Ferroplasma* and *Acidiplasma* are usually considered as heterotrophs, and require the presence of organic nutrients in the medium for growth [18,27,28]. Despite this, slight carbon dioxide fixation was demonstrated for some strains of the species *F. acidiphilum,* and possible genes of the “chimaeric pathway” for CO_2_ fixation were found in *F. acidarmanus* fer1 and *Ferroplasma* type I and II [29,30]. Thus, carbon dioxide may play some role in the growth of *Ferroplasma* representatives, but this aspect is poorly understood. The archaea of the genus *Cuniculiplasma* detected in the present work in the populations formed in a batch mode are heterotrophs [31]. The non-cultivated archaea “*Ca.* Carboxiplasma ferriphilum” (A-plasma group), described based on metagenomic data, is probably a heterotrophic iron-oxidizer [20].

Differences in carbon nutrition between acidophilic microorganisms oxidizing sulfide minerals result in inter-species interactions in the microbial population performing biooxidation on ores and concentrates. In natural ecosystems and abandoned mining ecosystems associated with the biooxidation of sulfide minerals, autotrophic acidophiles (*Acidithiobacillus*, *Leptospirillum*) produce organic compounds and facilitate the growth of heterotrophic acidophiles (*Ferroplasma*) [32,33]. Experiments on pyrite biooxidation by mixed cultures of different species of autotrophic and mixotrophic acidophiles also demonstrated the importance of these interspecies interactions for sulfide mineral oxidation. Okibe and Johnson [34] showed the crucial role of mutualistic interactions between different moderately thermophilic acidophiles [34]. The autotrophic sulfur oxidizer *A*. *caldus* and heterotrophic iron-oxidizer *Acidimicrobium ferrooxidans* were unable to leach pyrite with a high rate in pure culture. However, a mixed culture of these bacteria was capable of pyrite bioleaching. In this case, the autotrophic sulfur oxidizer *A*. *caldus* was incapable of pyrite leaching, and produced organic compounds, which in turn provided the growth of heterotrophic iron-oxidizer *Am. ferrooxidans*. Similar results have been previously reported by Bulaev [35]. It was shown that the mixotrophic iron- and sulfur-oxidizing bacterium S. *thermosulfidooxidans* VKMB1269^T^ was not able to oxidize pyrite in the absence of organic nutrients, while the presence of autotrophic sulfur oxidizer *A*. *caldus* in mixed culture provided the high activity of S. *thermosulfidooxidans* and a high rate of pyrite leaching.

This fact suggests that CO_2_ may be the sole carbon source, aiding in the activity of microbial populations performing the biooxidation of sulfide ores and concentrates, despite these populations including mixo- and heterotrophic species. Therefore, its effect on the growth of different acidophiles has been studied. MacLean et al. [36] studied the effects of CO_2_ concentration on the growth of several species of acidophiles including *S. thermosulfidooxidans*, *S. acidophilus*, *A. ferrooxidans*, and *A. caldus*. It was shown that the growth of studied strains of the genus *Sulfobacillus* depended on the concentration of CO_2_ in the air to a greater extent than the strains of the genus *Acidithiobacillus*, and CO_2_-enriched air provided higher growth rates and facilitated sulfur and ferrous iron oxidation by *Sulfobacillus* representatives. Thus, the growth and capacity for iron and sulfur biooxidation by some species of acidophiles depend on CO_2_ limitation.

Despite the potentially significant importance of understanding the effect of the carbon source on the biooxidation of sulfide ores and concentrates, there is limited information available on this subject. The reviews [3,19] postulate that CO_2_ is critically important for bioleaching processes, especially due to the low solubility of carbon dioxide in acidic solutions, and CO_2_ supply in bioleach reactors may be provided by impeller systems. Also, another review has summarized numerous works, which demonstrate that additional carbon dioxide supply may both enhance microbial growth and the biooxidation of ferrous iron [3]. Developers of BIOX^®^ technology recommend to use sulfide concentrates containing at least 2% carbonate to provide sufficient CO_2_ during biooxidation, or add limestone or CO_2_ into bioleach reactors as a carbon source [6]. Unfortunately, no data describing the effect of CO_2_ on biooxidation in BIOX^®^ plants have been published. Also, Gericke and co-authors [37] reported on the application of CO_2_ supply for the semi-industrial STRB of a nickel–cobalt concentrate, but they did not provide any comparative data on the effects of CO_2_ on bioleaching under different conditions.

Witne and Phillips [38] performed a series of bioleaching tests with copper concentrate in a batch mode, and studied the effects of different CO_2_ concentrations in air on copper extraction. It was shown that CO_2_-enriched air made it possible to increase the copper bioleaching rate and decrease the lag-phase of acidophilic microorganisms in bioleach reactors. In the work [39], the batch bioleaching of copper concentrate was performed using CO_2_-enriched air (0.5–2%). It was shown that CO_2_ supply provided a higher copper extraction rate (by ~1.5 times), while the total reached copper extraction differed insignificantly (87–90%). In the work [40], the effect of CO_2_-enriched (1%) air on the continuous STRB of cobalt pyrite concentrate was assessed. It was shown that additional CO_2_ supply was able to increase cobalt extraction from 65 to 90%.

Thus, several studies demonstrated the effects of CO_2_ supply on the bioleaching of different concentrates, and CO_2_ is considered a main source of carbon for microbial populations performing bioleaching. At the same time, some issues regarding the effects of carbon sources on STRB processes should be addressed. As STRB is successfully used at an industrial scale, further improvements, which may potentially facilitate the enhancing of biooxidation intensity, require comparative studies. Additionally, the impacts of such improvements should be investigated under various conditions to assess the feasibility of implementing various modifications to address the critical challenges of the process.

In our previous work [20], we studied the effects of CO_2_ on the continuous biooxidation of pyrite-arsenopyrite concentrate at different temperatures (40–45–50 °C). In this case, a temperature increase led to a decrease in the intensity of biooxidation, which is a problem for industrial biooxidation plants as a significant portion of OPEX is spent on cooling bioleach reactors [6]. The application of additional carbon dioxide as a carbon source made it possible to prevent the inhibition of biooxidation. Therefore, CO_2_ supply may be proposed as a solution to counteract the negative effects of temperature rise on biooxidation. Moreover, it is important to study the possibility of applying CO_2_ supply to avert other STRB problems, for example, to increase pulp density.

In the present work, we determined that CO_2_ used as an additional carbon source to alleviate the inhibition caused an increase in pulp density from S:L 1:10 to 1:5. In this case, it was possible to reach a high rate of arsenopyrite oxidation (89.8%). In our previous study, we showed that for the studied concentrate, arsenopyrite oxidation is crucial for further gold extraction, while the pyrite oxidation rate affected gold extraction to a lesser extent [20]. In contrast to the results obtained in the work [20], in this study, CO_2_ supply did not alleviate the effects of the increase in temperature. This may be explained by the fact that in a previous work, we adapted the microbial population in a batch mode to increasing temperatures [20]. Despite this, in this work, CO_2_ supply provided a lower rate of decrease in biooxidation activity under thermal stress. This suggests that in cases of short-term temperature rises (for example, due to a cooling system failure or an increase in ambient temperature), CO_2_ supplement may be an effective solution. 

The results on the microbial population analysis obtained in this work show that an increase in the pulp density did not lead to quality changes in microbial population composition (Figure 2 and Table S3), but a fraction of the bacteria of the genus *Acidithiobacillus* increased in comparison to the “normal conditions” and to the control reactor, which may be explained by the dependence of the growth of autotrophic *Acidithiobacillus* on CO_2_ supply [36]. In contrast, increasing temperature led to a significant change in the composition of the microbial population (Figure 2 and Table S3). In this case, CO_2_ supply led to an increase in the fraction of *Sulfobacillus*. This fact corresponds to the results shown in [36] and our previous work, when CO_2_ supply and increasing temperature led to an increase in the fraction of *Sulfobacillus* in the population.

Thus, the practical significance and novelty of the results obtained in the present work are as follows:

- The application of CO_2_ supply may decrease the negative effects of pulp density and temperature increase on the biooxidation of sulfide concentrate;
- An analysis of microbial populations formed after the shift in biooxidation conditions revealed patterns, corresponding to known data on properties of microorganisms;
- The results obtained in the present work may be partially explained based on known properties of microorganisms and patterns of biooxidation. At the same time, some phenomena observed require further analysis. CO_2_ supply significantly affected biooxidation only under “stressful conditions”;
- In the case of increasing temperature, the effects observed and differences between “normal” and “stressful conditions” can easily be explained. Carbon dioxide solubility decreases with increasing temperature [41]. Thus, increasing pulp temperature may lead to carbon dioxide shortages in the pulp and the inhibition of the growth of microorganisms and biooxidation. In the case of increasing temperature, additional carbon dioxide supply may partially alleviate the negative effect, which was shown in the present study and our previous works [20,21,22,23]. Moreover, a shift in the composition of microbial population due to increasing temperature also may be explained, since *Sulfobacillus* representatives may show temperature optima at temperatures around 50 °C and depend on CO_2_ concentration [18,36]. Increasing temperature led to the elimination of other microorganisms, while CO_2_ supply supported the growth of *Sulfobacillus* representatives, which led to an increase in their proportion in the population, as well as in the total cell number in the population, which in turn led to an increase in biooxidation rate (in comparison to the control at 50 °C);
- In contrast, the mechanisms of the effects of pulp density increase on biooxidation are less well understood. In general, numerous studies have demonstrated that increases in pulp density may adversely the affect biooxidation rate [3,6,42,43,44,45]. From one point of view, a minimum sulfide sulfur concentration of approximately 6% is usually required to promote the growth and activity of microorganisms [6]. At the same time, an increase in pulp density leads to the inhibition of the biooxidation of sulfide concentrates of various compositions, and concentrates with higher sulfide sulfur grades should be oxidized at lower pulp densities [6,42]. The review [42] proposed several different mechanisms of the adverse effects of high pulp density on bioleach reactor population, including increasing toxic ions concentration, inhibiting gas mass transfer (i.e., decreasing oxygen and carbon dioxide availability), the mechanical damage of cells, the formation of secondary precipitates (jarosite), and decreasing Eh (which in turn is crucial for pyrite bioleaching [46]). As was shown in the present study, decreasing carbon dioxide availability may be one of the main factors causing biooxidation inhibition at high pulp density, while additional carbon dioxide supply may neutralize this harmful effect. The effect of increasing pulp density and carbon dioxide supply on the microbial population cannot be unambiguously interpreted based on the results of the present work. Additional CO_2_ supply led to an increase in total cell number in the population, which in turn may result in increasing biooxidation rate. Also, carbon dioxide supply led to an increase in the proportion of the bacteria of the genus *Aciditihiobacillus*. This change in microbial population cannot be directly considered as the main reason for the increasing sulfide mineral oxidation rates. Among known representatives of the genus *Acidithiobacillus*, only the moderately thermophilic sulfur oxidizer *A*. *caldus* may be active under biooxidation conditions (at 40–50 °C) [18]. However, sulfur oxidizers may be less effective in sulfide mineral oxidation in comparison to iron-oxidizing microorganisms (especially pyrite, the biooxidation of which requires ferrous iron oxidation and a high Fe^3+^/Fe^2+^ ratio [46]). At the same time, the sulfur-oxidizing autotroph *A*. *caldus* may supply iron-oxidizing heterotrophic acidophiles *Ferroplasma*, which also predominated in the population [32,33]. Thus, carbon dioxide supply at a higher pulp density may directly provide the growth of sulfur-oxidizing autotroph *A*. *caldus*, which in turn favorably affects the iron-oxidizing heterotroph *Ferroplasma*. These phenomena (inhibition of gas mass transfer at high pulp density, as well as interspecies interactions between acidophilic auto- and heterotrophs) may be the reasons for the positive effects of carbon dioxide supply on biooxidation under “stressful conditions”;
- Explanations of carbon dioxide’s effects on biooxidation at high temperature and pulp density may also clarify the possible causes of the weak effect of CO_2_ supply under “normal conditions”. It may be assumed that at lower temperature and pulp density, gas mass transfer was sufficient to provide the activity of microbial population using air as the carbon dioxide source. It should also be noted that pyrite biooxidation was weakly affected by CO_2_ supply even under “normal conditions”, while the average total cell number in CO_2_-supplied reactors was higher than those in control reactors. This suggests that under “normal conditions”, carbon dioxide supply also affected biooxidation to a lesser extent in comparison to “stressful conditions”, but the limited availability of carbon source was not a crucial factor affecting the microbial population.

## 5. Conclusions

In the present work, we studied the possibility of counteracting the negative effects of unfavorable conditions (increasing pulp density and temperature) on the biooxidation of a pyrite-arsenopyrite concentrate in laboratory-scale stirred tank reactors using additional carbon supply in the form of CO_2_. In the control experiment, biooxidation was performed under “normal conditions”: temperature 40 °C, pulp density (solid to liquid ratio, S:L) 1:0, residence time 5 days. In this case, under “normal conditions”, additional carbon dioxide supply insignificantly affected the biooxidation rate and composition of the microbial population of biooxidation reactors. 

In this case of “stressful conditions”, either temperature or pulp density were increased (up to 50 °C and S:L to 1:5, respectively), which provided unfavorable conditions for biooxidation and led to a decrease in the biooxidation rate. Under “stressful conditions”, additional carbon dioxide supply affected biooxidation to a greater extent and made it possible to increase both pyrite and arsenopyrite biooxidation rates. In the case of increasing pulp density, CO_2_ supply made it possible to reach a high rate of arsenopyrite biooxidation. The analysis of microbial populations showed that increasing pulp density and CO_2_ supply did not change their composition significantly. In contrast, at increasing temperature (50 °C), CO_2_ supply affected biooxidation to a lesser extent. Despite this, in this work, CO_2_ supply provided a lower rate of decrease in biooxidation activity under thermal stress. In the case of increasing temperature, additional carbon dioxide supply also changed the microbial population’s composition and led to the predominance of the bacteria of the genus *Sulfobacillus*.

In the present work, the results were obtained on a laboratory scale. However, it can be assumed that the approach studied in our work may be promising for application on an industrial scale, as laboratory tests were performed under conditions close to those used in industrial-scale plants. Moreover, it is known that industrial scale bioleach reactors may be equipped with CO_2_ supply systems. The increase in robustness of the biooxidation process is an urgent issue. Therefore, further tests on the pilot and industrial scale are required for the further development of the method proposed.

## Figures and Tables

**Table 1:** Averaged values of pulp liquid phase parameters during biooxidation in “normal conditions” (average values ± SD, *n* = 8).

Bioreactor	Pulp Density (S:L)	T, °C	CO_2_	pH	Eh, mV	Fe^3+^, g/L	Fe^2+^, g/L	Fe_tot_, g/L	As, g/L	N × 10^7^, Cell/mL
1	1:10	40	+	0.90 ± 0.06	776 ± 13	26.98 ± 1.95	0.09 ± 0.12	27.07 ± 1.91	6.60 ± 0.92	198 ± 65
2	1:10	40	−	0.98 ± 0.03	783 ± 18	26.28 ± 1.85	0.14 ± 0.30	26.42 ± 1.74	6.58 ± 0.68	157 ± 47
3	1:10	40	+	0.94 ± 0.07	785 ± 9	27.00 ± 1.61	0.04 ± 0.10	27.04 ± 1.61	6.41 ± 0.49	181 ± 72
4	1:10	40	−	0.92 ± 0.06	775 ± 14	23.67 ± 2.11	0.16 ± 0.20	23.83 ± 2.00	5.50 ± 0.32	124 ± 25
Average	1:10	40		0.96 ± 0.03	780 ± 5	25.98 ± 1.58	0.11 ± 0.06	26.09 ± 1.54	6.27 ± 0.52	165 ± 32

**Table 2:** Averaged values of pulp liquid phase parameters during biooxidation under “stressful conditions” (average values ± SD, *n* = 5).

Bioreactor	Pulp Density (S:L)	T, °C	CO_2_	PH	Eh, mV	Fe^3+^, g/L	Fe^2+^, g/L	Fe_tot_, g/L	As, g/L	N × 10^7^, Cell/mL
1	1:5	40	+	0.86 ± 0.06	733 ± 10	25.20 ± 1.97	0.95 ± 0.46	26.15 ± 1.58	11.66 ± 0.33	223 ± 40
2	1:5	40	−	1.09 ± 0.09	668 ± 13	15.18 ± 2.86	4.09 ± 0.96	19.26 ± 2.13	9.50 ± 0.42	150 ± 49
3	1:5	50	+	1.09 ± 0.07	684 ± 17	10.36 ± 1.94	1.51 ± 1.35	11.87 ± 1.45	4.23 ± 2.24	12 ± 8
4	1:5	50	−	1.30 ± 0.06	663 ± 3	4.79 ± 1.11	2.58 ± 0.62	7.36 ± 1.42	2.71 ± 1.53	7 ± 4

**Table 3:** Summary of biooxidation results.

Mode	Bioreactor	Pulp Density (S:L)	T, °C	CO_2_ Supply	Residue Mass Yield, %	Oxidation, %		CaCO_3_, kg/t of the Concentrate
						Arsenopyrite (FeAsS)	Pyrite (FeS_2_)	
“Normal conditions”	1	1:10	40	+	59.7	97.4 ± 0.2	81.0 ± 0.3	112.5 ± 69.4
	2	1:10	40	−	62.7	97.1 ± 0.2	75.6 ± 0.3	131.3 ± 53.0
	3	1:10	40	+	60.9	97.1 ± 0.2	81.1 ± 0.3	143.8 ± 62.3
	4	1:10	40	−	57.9	97.2 ± 0.2	74.3 ± 0.3	156.3 ± 67.8
	Average for “Normal conditions” (CO_2_ supply)	1:10	40	+	60.3	97.3 ± 0.2	81.1 ± 0.3	128.1 ± 22.1
	Average for “Normal conditions” (without CO_2_ supply)	1:10	40	−	60.3	97.2 ± 0.2	75.00 ± 0.3	143.8 ± 17.7
	Average for “Normal conditions”	1:10	40		60.3	97.2 ± 0.2	78.0 ± 0.3	135.9 ± 18.7
“Stressful conditions”	1	1:5	40	+	65.5	89.8 ± 0.5	45.7 ± 0.5	100.0 ± 141.4
	2	1:5	40	−	79.4	69.9 ± 0.9	23.5 ± 0.7	0 ± 0
	3	1:10	50	+	68.3	63.9 ± 1.1	28.8 ± 0.6	0 ± 0
	4	1:10	50	−	81.7	58.6 ± 1.2	26.5 ± 0.7	0 ± 0

## Data Availability

The raw data generated from the 16S rRNA gene profiling are accessible via the BioProject accession number PRJNA1166071.

## Supplementary Materials

The following supporting information can be downloaded at: https://www.mdpi.com/article/10.3390/microorganisms12122463/s1, Figure S1: XRD analysis of the concentrate and biooxidation products; Figure S2: Changes in the concentrations (g/L) of Fe^3+^ (a and c) and Fe^2+^ (b and d) ions during biooxidation; Table S1: Sulfide iron, arsenic, and sulfur content in the concentrate and biooxidation residue calculated according to XRF analysis; Table S2: Chemical composition of the concentrate and biooxidation residues according to XRF analysis; Table S3: An analysis of the microbial populations of bioleach reactors formed under different conditions—a proportion of the 16S rRNA gene fragment, %.
