# Supplementary material for: Biooxidation of a Pyrite-Arsenopyrite Concentrate Under Stressful Conditions
Source: Microorganisms. 2024 Nov 29;12(12):2463. doi: 10.3390/microorganisms12122463 (PMC11678047; doi:10.3390/microorganisms12122463)
Supplement: Supplementary file 1 [file microorganisms-12-02463-s001.zip › microorganisms-3304140-supplementary.pdf]

Biooxidation of a Pyrite-Arsenopyrite Concentrate Under Stressful Conditions

Aleksandr Bulaev *, Alena Artykova, Anna Diubar, Aleksandr Kolosoff, Vitaliy Melamud, Tatiana Kolganova, Alexey Beletsky and Andrey Mardanov

Research Center of Biotechnology, Russian Academy of Sciences, 119071 Moscow, Russia; alena.artykov@gmail.com (A.A.); annadbr1@yandex.ru (A.D.); alexander_thechemist_kolosoff@mail.ru (A.K.); vmelamud.inmi@yandex.ru (V.M.); moldiag@biengi.ac.ru (T.K.); mortu@yandex.ru (A.B.)

* Correspondence: bulaev-inmi@yandex.ru; Tel.: +7-499-135-04-21

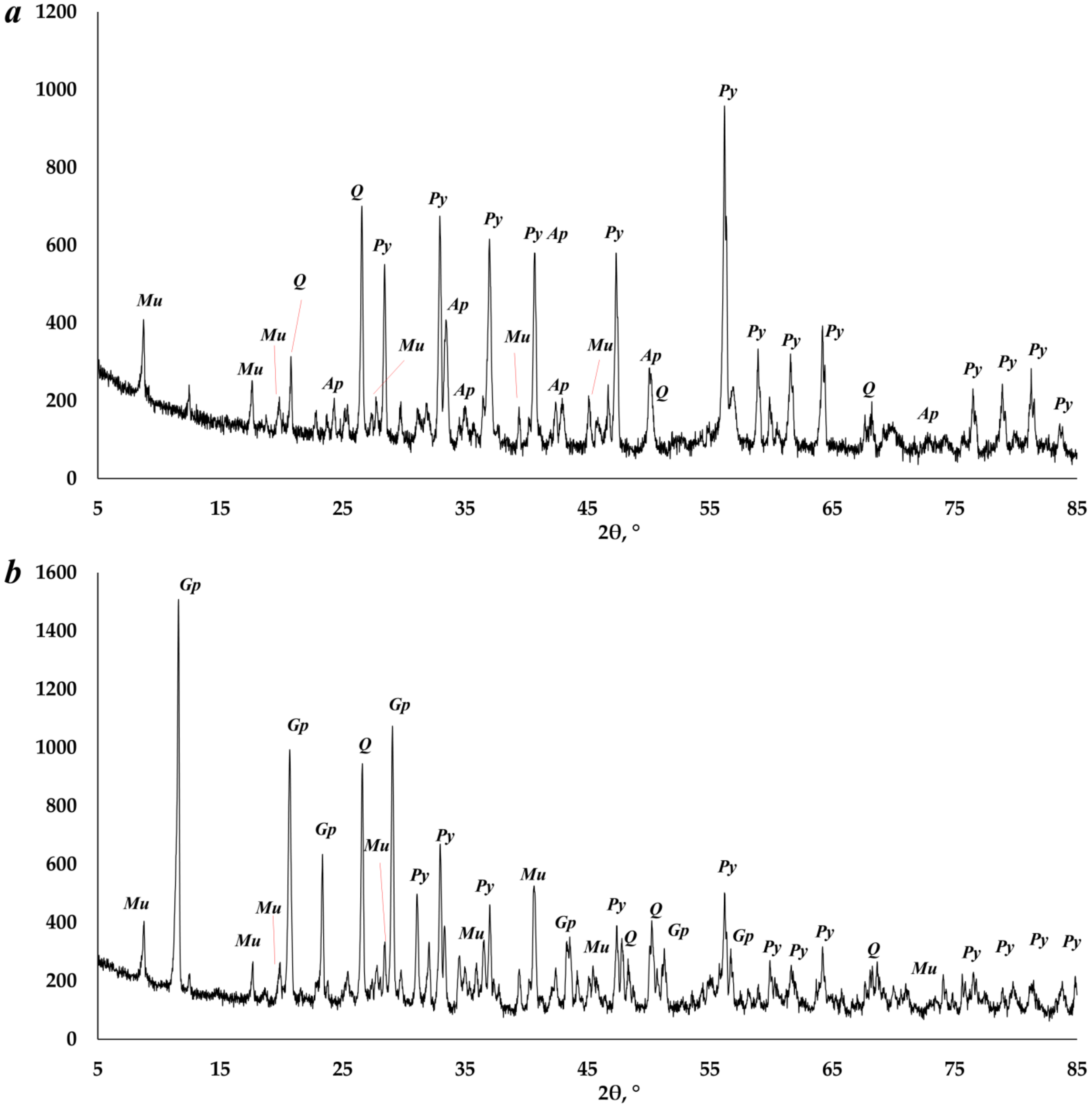

a
1200
1000
800
600
400
200
0
5
15
25
35
45
55
65
75
85
2θ, °
Mu
Q
Mu
Mu
Ap
Q
Py
Mu
Py
Ap
Ap
Py
Mu
Py Ap
Ap
Mu
Py
Ap
Q
Py
Py
Py
Py
Q
Ap
Py
Py
Py
Py
b
1600
1400
1200
1000
800
600
400
200
0
5
15
25
35
45
55
65
75
85
2θ, °
Gp
Gp
Gp
Q
Gp
Mu
Py
Py
Py
Mu
Mu
Mu
Mu
Gp
Py
Q
Q
Gp
Mu
Py
Gp
Py
Py
Py
Q
Mu
Py
Py
Py
Py

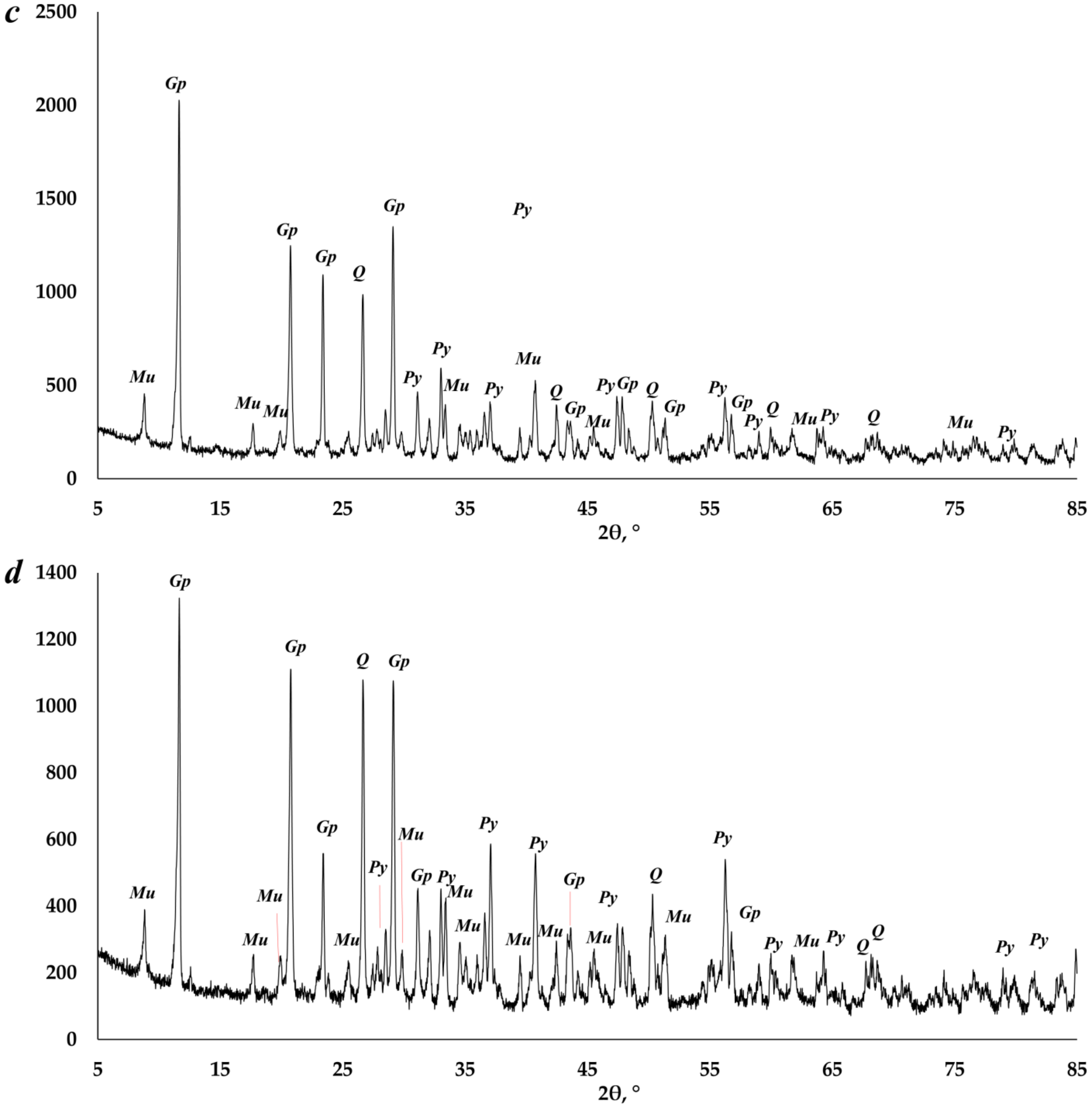
c
2θ, °
Gp
Q
Py
Mu
d
2θ, °
Gp
Q
Py
Mu

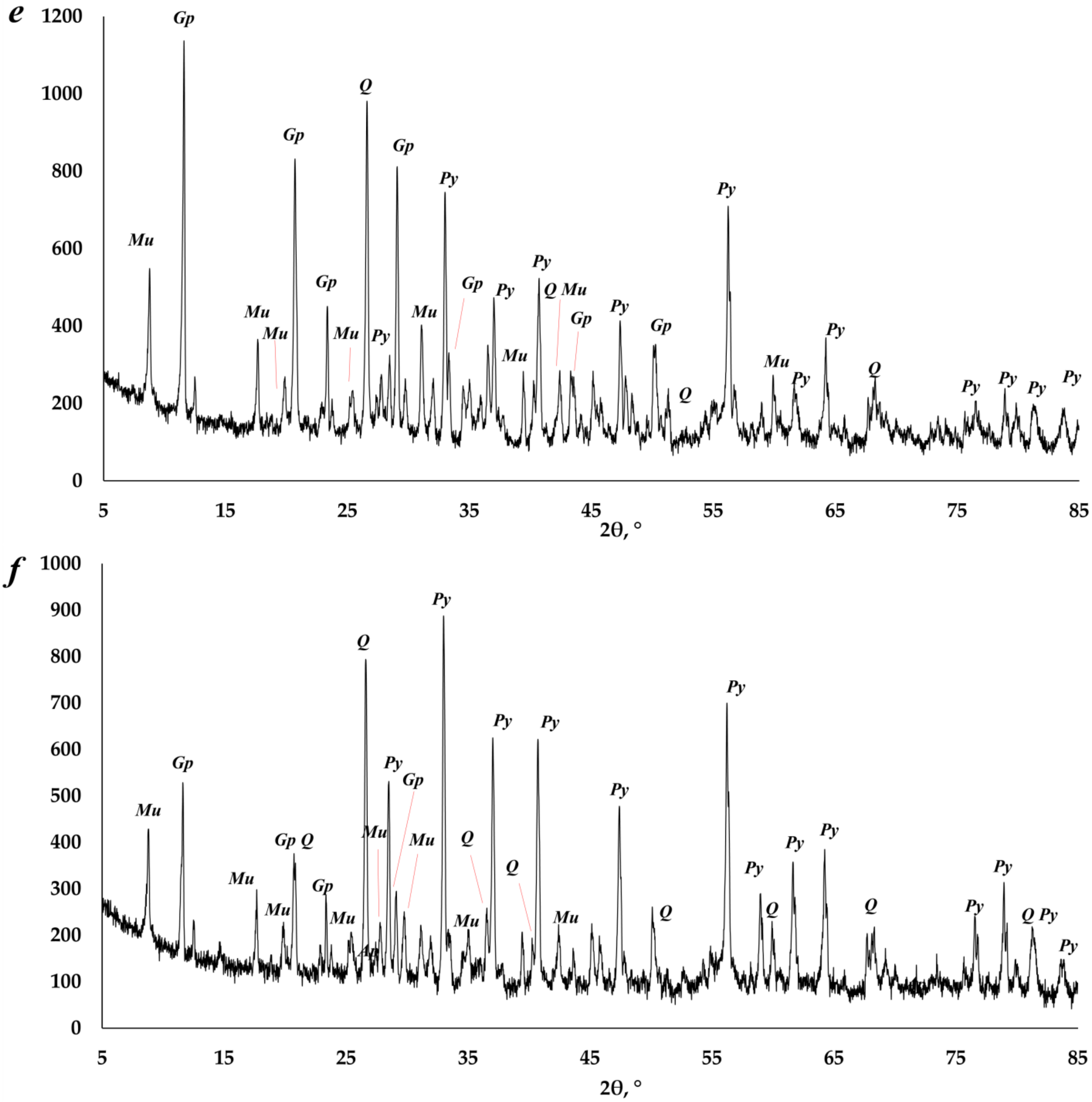
e
1200
1000
800
600
400
200
0
5
15
25
35
45
55
65
75
85
2θ, °
Gp
Q
Gp
Gp
Py
Mu
Py
Mu
Gp
Gp
Py
Q Mu
Mu
Mu
Py
Mu
Mu
Gp
Py
Gp
Q
Mu
Py
Py
Q
Py
Py
Py
Py
f
1000
900
800
700
600
500
400
300
200
100
0
5
15
25
35
45
55
65
75
85
2θ, °
Py
Q
Py
Py
Py
Gp
Py
Mu
Py
Gp
Gp Q
Mu
Mu
Q
Py
Q
Mu
Py
Py
Mu
Gp
Q
Mu
Mu
Q
Q
Q
Py
Py
Q Py
Py

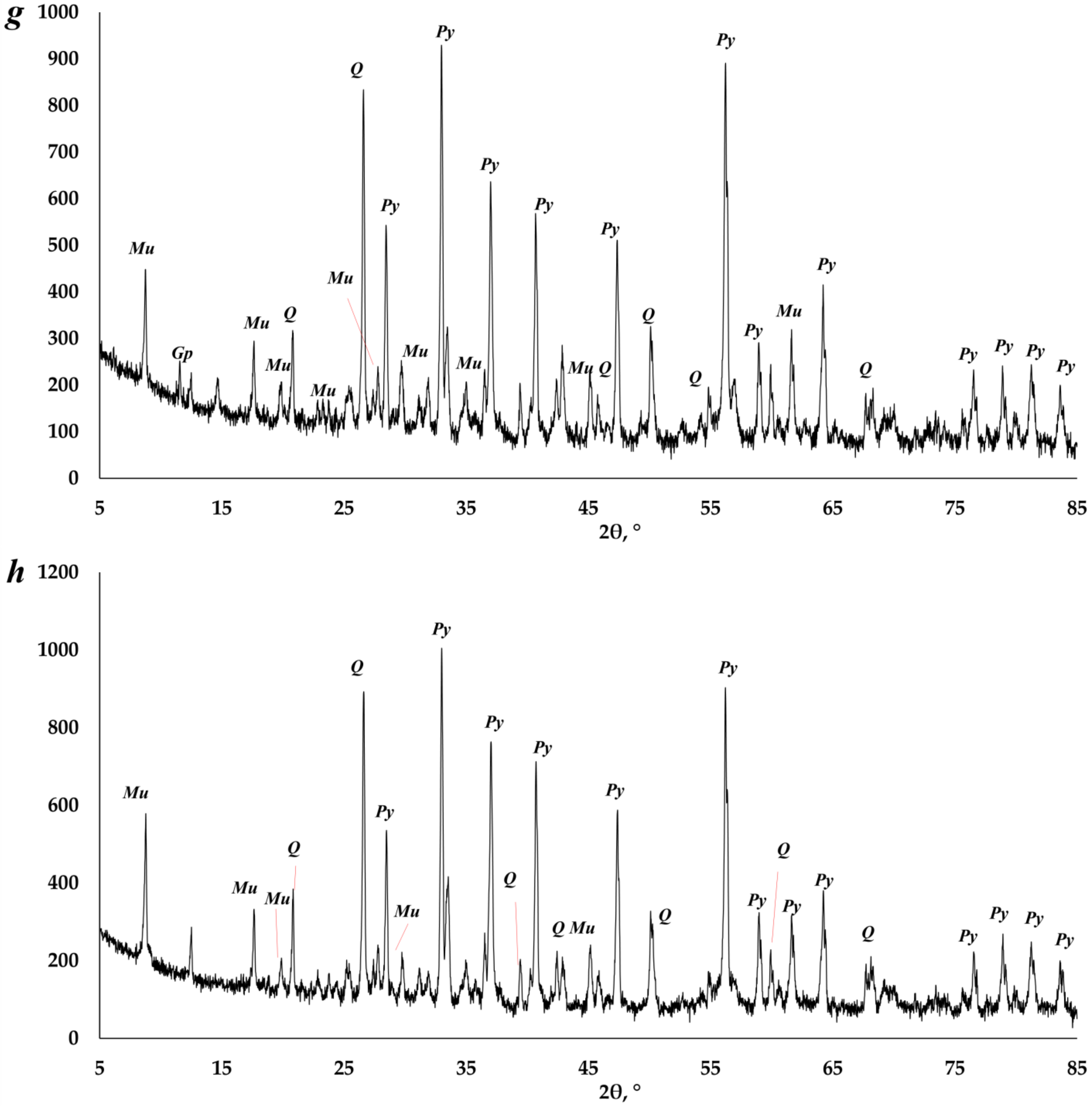
g
h
2θ, °
Mu
Gp
Q
Py

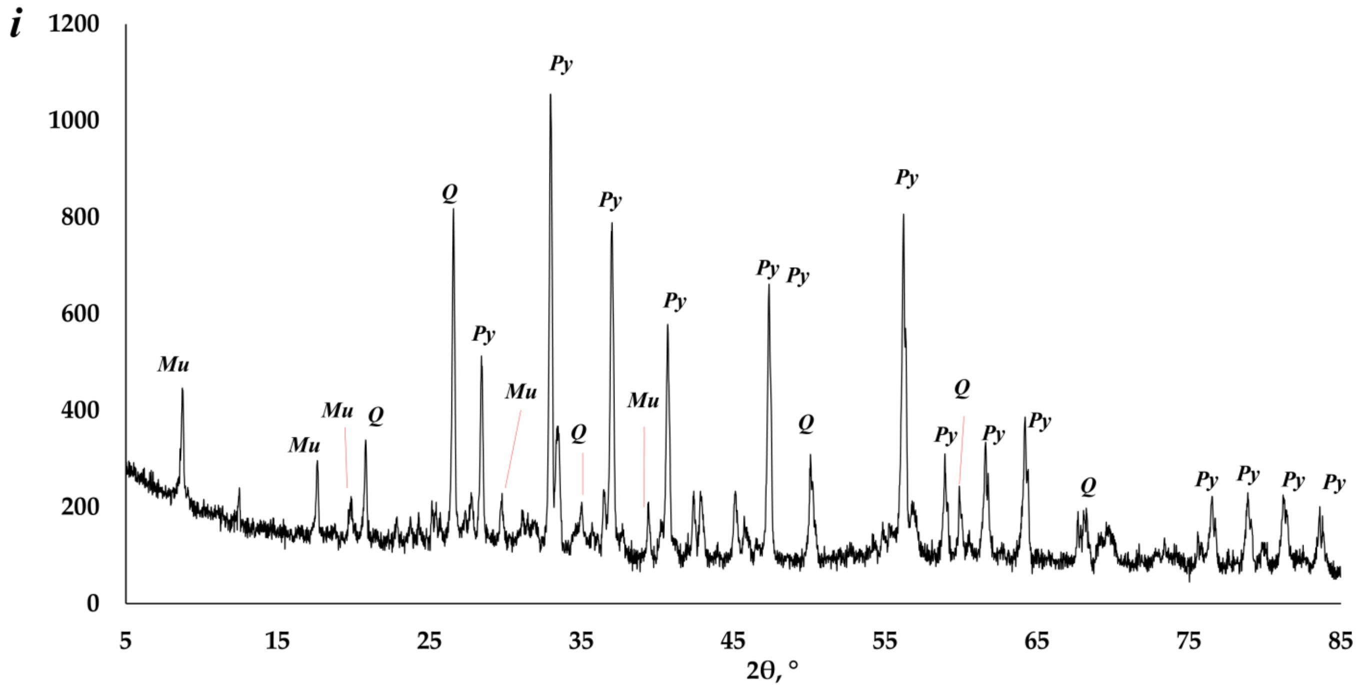

Figure S1. XRD analysis of the concentrate and biooxidation products (Rigaku Rotaflex RU-200 D/MAX-RC (Rigaku, Japan), Cu-Kα); *Ap* – arsenopyrite, *Gp* – gypsum, *Q* – quartz, *Mu* – muscovite, *Py* – pyrite; a – concentrate, b – biooxidation residue (reactor 1, “Normal conditions”, CO_2 supply), c – biooxidation residue (reactor 2, “Normal conditions”), d – biooxidation residue (reactor 3, “Normal conditions”, CO_2 supply), e – biooxidation residue (reactor 4, “Normal conditions”), f – biooxidation residue (reactor 1, “Stressful conditions” – S : L 1 : 5, CO_2 supply), g – biooxidation residue (reactor 2, “Stressful conditions” – S : L 1 : 5), h – biooxidation residue (reactor 3, “Stressful conditions” – 50°C, CO_2 supply), i – biooxidation residue (reactor 4, “Stressful conditions” – 50°C).

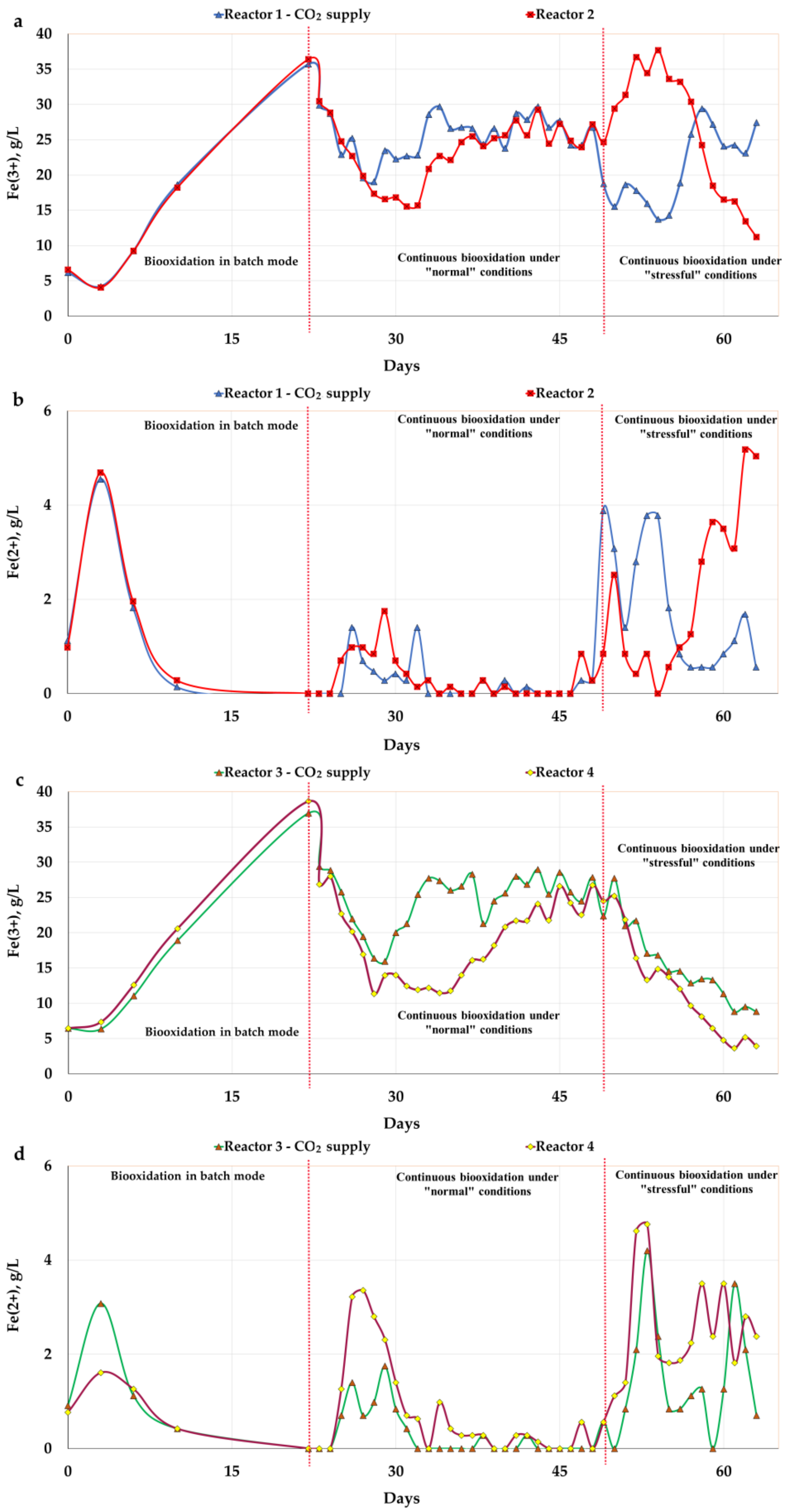

Figure S2. Changes in the concentrations (g/L) of Fe^{3+} (a and c) and Fe^{2+} (b and d) ions during biooxidation.

Table S1. Sulfide iron, arsenic, and sulfur content (%) in the concentrate and biooxidation residue calculated according to XRF analysis.

Sample	Mode	Bioreactor	Pulp density (S:L)	T, °C	CO_2 supply	Fe_s	As_s	S_s
Concentrate	-	-	-	-	-	26.9± 0.3	6.0± 0.1	25.1± 0.2
Bio-oxidation residue	"Normal conditions"	1	1 : 10	40	+	7.6± 0.1	0.2± 0.02	6.9± 0.1
		2	1 : 10	40	-	8.5± 0.1	0.3± 0.02	9.3± 0.1
		3	1 : 10	40	+	8.3± 0.1	0.3± 0.02	6.0± 0.1
		4	1 : 10	40	-	11.0± 0.1	0.3± 0.02	9.2± 0.12
	"Stressful conditions"	1	1 : 5	40	+	20.5± 0.2	0.9± 0.1	17.8± 0.2
		2	1 : 5	40	-	23.1± 0.3	2.3± 0.1	22.8± 0.2
		3	1 : 10	50	+	26.4± 0.3	3.2± 0.1	24.1± 0.2
		4	1 : 10	50	-	23.7± 0.3	3.1± 0.1	20.3± 0.2

Table S2. Chemical composition (element content, %) of the concentrate and biooxidation residues according to XRF analysis.

№	Element	Concentrate				Reactor 1 (40 °C, S:L 1:10, CO_2)				Reactor 2 (40 °C, S:L 1:10)				Reactor 3 (40 °C, S:L 1:10, CO_2)				Reactor 4 (40 °C, S:L 1:10)			
		Initial sample		HCl treatment*		Initial sample		HCl treatment*		Initial sample		HCl treatment*		Initial sample		HCl treatment*		Initial sample		HCl treatment*	
		content	SD	content	SD	content	SD	content	SD	content	SD	content	SD	content	SD	content	SD	content	SD	content	SD
1	Fe	35.3000	0.2400	29.7100	0.3000	8.8600	0.1600	13.9500	0.2100	8.7300	0.1600	15.8600	0.2100	8.6300	0.1600	13.3600	0.2000	15.0600	0.2100	17.7200	0.2300
2	Sx	31.8700	0.2300	27.6500	0.2500	18.4300	0.2200	12.7300	0.1700	18.6000	0.2200	17.2100	0.2000	17.8000	0.2200	9.7000	0.1500	20.5100	0.2300	14.8600	0.1900
3	Si	16.2000	0.1800	15.2500	0.1800	13.9800	0.1800	28.3500	0.2400	11.8500	0.1700	31.4300	0.2400	12.1600	0.1700	30.1400	0.2400	16.9300	0.1900	28.7900	0.2300
4	As	7.0500	0.1400	6.6700	0.1400	0.4980	0.0300	0.4800	0.0320	0.4670	0.0290	0.5120	0.0350	0.5370	0.0310	0.4650	0.0310	0.5860	0.0370	0.4780	0.0340
5	Al	6.9100	0.1300	6.6600	0.1200	5.6400	0.1200	11.4300	0.1600	4.7800	0.1100	11.1600	0.1600	4.8400	0.1100	11.8700	0.1600	6.7700	0.1300	10.9600	0.1600
6	K	1.3200	0.0600	1.0900	0.0500	1.1200	0.0500	2.7400	0.0800	1.0900	0.0500	2.8500	0.0800	1.2000	0.0500	2.6100	0.0800	1.6500	0.0600	2.5200	0.0800
7	Ti	0.3670	0.0200	0.3470	0.0180	0.4530	0.0230	0.9710	0.0490	0.4170	0.0210	1.0600	0.0500	0.3650	0.0180	0.8880	0.0440	0.5510	0.0280	0.8860	0.0440
8	Na	0.3280	0.0740	n.d.	n.d.	0.7350	0.0380	0.2770	0.0640	0.2710	0.0530	1.1400	0.0500	0.1890	0.0530	0.6870	0.0560	0.2910	0.0570	0.7010	0.0620
9	Mg	0.2620	0.0310	n.d.	n.d.	0.4380	0.0220	0.1110	0.0230	0.1410	0.0220	0.6120	0.0310	0.1550	0.0220	0.2640	0.0230	0.2400	0.0220	0.2850	0.0240
10	Cu	0.1110	0.0150	0.0310	0.0100	0.0088	0.0042	0.0200	0.0057	0.0177	0.0050	0.0128	0.0063	0.0099	0.0045	0.0143	0.0047	0.0398	0.0069	0.0312	0.0074
11	Ca	0.0680	0.0100	0.0076	0.0045	9.8400	0.1600	0.1440	0.0110	9.8600	0.1600	0.1850	0.0130	9.2200	0.1500	0.1910	0.0130	6.3000	0.1200	0.0640	0.0081
12	Co	0.0672	0.0096	0.0394	0.0089	0.0203	0.0049	0.0235	0.0053	0.0182	0.0047	0.0180	0.0058	0.0138	0.0044	0.0193	0.0047	0.0236	0.0053	0.0278	0.0063
13	Px	0.0550	0.0100	0.0147	0.0088	0.0497	0.0079	n.d.	n.d.	0.0176	0.0061	n.d.	n.d.	0.0489	0.0072	0.0188	0.0085	0.0191	0.0071	0.0092	0.0083
14	Ni	0.0430	0.0100	0.0453	0.0095	n.d.	n.d.	n.d.	n.d.	0.0184	0.0042	n.d.	n.d.	0.0182	0.0043	0.0316	0.0051	0.0117	0.0039	0.0313	0.0064
15	Zn	0.0410	0.0110	0.0087	0.0086	0.0081	0.0040	0.0246	0.0058	0.0086	0.0041	0.0149	0.0062	n.d.	n.d.	0.0123	0.0044	0.0159	0.0048	n.d.	n.d.

Table S2. Chemical composition of the concentrate and biooxidation residues according to XRF analysis (***continuation***).

№	Element	Reactor 1 (40 °C, S:L 1:5, CO_2)		Reactor 2 (40 °C, S:L 1:5)		Reactor 3 (50 °C, S:L 1:10, CO_2)		Reactor 4 (50 °C, S:L 1:10)	
		Initial sample	HCl treatment*	Initial sample	HCl treatment*	Initial sample	HCl treatment*	Initial sample	HCl treatment*

		content	SD	content	SD	content	SD	content	SD	content	SD	content	SD	content	SD	content	SD
1	Fe	24.9800	0.2700	23.9300	0.2700	32.8500	0.3000	25.6500	0.2900	31.2800	0.3100	28.3200	0.2900	30.4800	0.3100	27.5000	0.3000
2	Sx	25.1200	0.2400	20.7900	0.2200	32.0900	0.2600	25.3500	0.2400	26.6200	0.2400	25.8900	0.2400	22.9900	0.2300	23.5200	0.2300
3	Si	19.1900	0.2000	22.5300	0.2100	17.9900	0.1900	15.4700	0.1800	18.6200	0.2000	19.0100	0.2000	17.0500	0.1900	18.9900	0.2000
4	As	1.5900	0.0700	1.1000	0.0500	4.0300	0.1200	2.5400	0.0800	4.0200	0.1100	3.4200	0.1000	6.7000	0.1400	3.5500	0.1000
5	Al	7.9500	0.1400	9.2300	0.1400	7.5500	0.1300	6.8300	0.1300	7.6000	0.1300	6.8400	0.1300	6.6000	0.1200	6.8600	0.1300
6	K	1.7900	0.0700	1.9000	0.0700	1.4800	0.0600	1.3300	0.0600	1.4800	0.0600	1.3700	0.0600	1.4600	0.0600	1.4400	0.0600
7	Ti	0.5450	0.0270	0.6720	0.0340	0.4530	0.0230	0.3730	0.0190	0.4440	0.0220	0.4120	0.0210	0.4520	0.0230	0.3640	0.0180
8	Na	0.3480	0.0650	0.1520	0.0640	n.d.	n.d.	0.3090	0.0690	n.d.	n.d.	0.0900	0.0590	n.d.	n.d.	0.1000	0.0610
9	Mg	0.2250	0.0270	0.2360	0.0260	0.2700	0.0330	0.1850	0.0260	0.1630	0.0320	0.1320	0.0240	0.2390	0.0360	0.0830	0.0240
10	Cu	0.0820	0.0110	0.0585	0.0099	0.0990	0.0140	0.0394	0.0090	0.0710	0.0120	0.0452	0.0098	0.0590	0.0100	0.0362	0.0094
11	Ca	2.2400	0.0700	0.0304	0.0064	1.1900	0.0500	0.0098	0.0045	0.0206	0.0059	0.0273	0.0071	0.0348	0.0057	0.0086	0.0049
12	Co	0.0303	0.0068	0.0450	0.0078	0.0337	0.0080	0.0340	0.0073	0.0330	0.0083	0.0278	0.0072	0.0441	0.0076	0.0278	0.0073
13	Px	0.0540	0.0096	0.0244	0.0078	0.0300	0.0100	0.0166	0.0072	0.0339	0.0089	0.0513	0.0086	0.0468	0.0096	0.0136	0.0074
14	Ni	0.0190	0.0061	0.0217	0.0065	0.0332	0.0080	0.0131	0.0061	0.0366	0.0087	0.0465	0.0084	0.0807	0.0097	0.0271	0.0074
15	Zn	n.d.	n.d.	n.d.	n.d.	0.0260	0.0085	0.0156	0.0071	0.0238	0.0090	0.0253	0.0081	0.0149	0.0064	0.0054	0.0069

*Samples were treated with 10%HCl for 30 min at 100°C for removal oxide iron and iron minerals, sulfide iron, arsenic, and sulfur contents were calculated according to formula:

$$C_s(\%) = C_{HCl}(\%) \times RE_{HCl}(\%) \times 0.01,$$

where C_s is sulfide element content; $C_{HCl}(\%)$ is element content in the sample treated with 10% HCl; $RE_{HCl}(\%)$ is mass residue yield (%) after treatment with 10% HCl [24].

Table S3. An analysis of the microbial populations of bioleach reactors formed under different conditions – a proportion of the 16S rRNA gene fragment, %.

**Group of microorganisms**	**Inoculum**	**Batch conditions (40 °C)**					**Continuous - Mode 1 ("Normal" conditions - 40 °C, S:L 1:10)**					**Continuous - Mode 2 ("Stressful" conditions)**			
		**Reactor 1 (CO_2)**	**Reactor 2**	**Reactor 3 (CO_2)**	**Reactor 4**	**Average**	**Reactor 1 (CO_2)**	**Reactor 2**	**Reactor 3 (CO_2)**	**Reactor 4**	**Average**	**Reactor 1 (40 °C, S:L 1:5, CO_2)**	**Reactor 2 (40 °C, S:L 1:5)**	**Reactor 3 (50 °C, S:L 1:10, CO_2)**	**Reactor 4 (50 °C, S:L 1:10)**
Acidithiobacillus	13.10	14.23	40.46	26.74	9.50	22.73±13.88	10.89±2.96	13.94±7.47	14.40±0.66	37.06 ±6.17	19.07±11.84	46.28±56.26	17.31±8.70	3.98±3.83	37.96±51.88
Acidiferrobacter	0.00	0.00	0.00	0.00	0.00	0.00±0.00	0.00±0.00	0.00±0.00	0.00±0.00	0.21±0.30	0.05±0.15	0.01±0.01	0.00±0.00	0.00±0.00	0.00±0.00
Acidiphilium	0.00	0.00	0.00	0.00	0.00	0.00±0.00	0.00±0.00	0.00±0.00	0.00±0.00	0.21±0.29	0.05±0.15	0.00±0.00	0.00±0.00	0.00±0.00	0.00±0.00
Sulfobacillus	85.60	0.15	1.59	0.15	0.03	0.48±0.74	3.37±3.74	1.09±1.08	1.70±0.55	1.61±0.14	1.94±1.75	0.50±0.62	0.02±0.02	91.06±6.52	47.51±65.55
Leptospirillum	0.00	0.16	0.22	0.08	0.09	0.14±0.07	0.01±0.01	0.00±0.00	0.03±0.01	0.21±0.08	0.06±0.09	0.04±0.05	0.05±0.05	0.30±0.32	2.08±2.89
Ferrimicrobium	0.00	0.00	0.00	0.00	0.00	0.00±0.00	0.00±0.00	0.00±0.00	0.00±0.00	0.00±0.00	0.00±0.00	0.00±0.00	0.00±0.00	0.00±0.00	0.03±0.04
Ferroplasma	1.29	79.38	50.47	67.05	67.02	65.98±11.87	85.57±6.59	84.87±8.66	83.80±0.14	60.61 ±5.33	78.71±12.09	53.11±56.96	82.40±8.78	1.39±0.48	6.54±8.94
Cuniculiplasma	0.00	3.04	4.83	3.87	19.08	7.71±7.62	0.14±0.05	0.04±0.06	0.01±0.00	0.00±0.00	0.05±0.07	0.02±0.03	0.13±0.03	0.29±0.39	0.02±0.02
"*Ca.* Carboxiplasma ferriphilum"	0.00	3.02	2.26	2.04	4.28	2.90±1.01	0.02±0.03	0.06±0.05	0.05±0.03	0.04±0.04	0.04±0.03	0.02±0.03	0.10±0.05	2.78±2.11	0.08±0.12
Thermoplasmata uncultured	0.00	0.00	0.00	0.00	0.00	0.00±0.00	0.00±0.00	0.00±0.00	0.00±0.00	0.00±0.00	0.00±0.00	0.00±0.00	0.00±0.00	0.00±0.00	0.04±0.05
Other microorganisms	0.00	0.02	0.17	0.07	0.00	0.07±0.08	0.00±0.00	0.00±0.00	0.00±0.00	0.05±0.07	0.01±0.04	0.02±0.03	0.00±0.00	0.20±0.01	5.77±7.76
